# Castor Oil-Based Polyurethane Resin for Low-Density Composites with Bamboo Charcoal

**DOI:** 10.3390/polym10101100

**Published:** 2018-10-05

**Authors:** Yi-Chun Chen, Wei Tai

**Affiliations:** Department of Forestry, National Chung Hsing University, Taichung 402, Taiwan; davidtai2004@yahoo.com.tw

**Keywords:** adhesive, bamboo charcoal, castor oil, composites, polyurethane

## Abstract

Polyurethane (PU) foam adhesives were prepared from castor oil as a polyol with isocyanate poly(4,4’-methylene diphenyl isocyanate) (PMDI) using a solvent-free process. The NCO/OH molar ratio used for the preparation of PU foams was 1.5. Water, organosiloxane and dibutyltin dilaurate were used as the blowing agent, surfactant and catalyst, respectively. The ratio of blowing agent and catalyst were adjusted to optimize the properties. The results show that PU foam prepared with 4 wt % of castor oil catalyst and blowing agent has minimal water absorption and maximal volume expansion in the PU foams. FT-IR analysis shows that a urethane bond was formed by the hydroxyl group of castor oil and the –NCO group of isocyanate PMDI. More blowing agent and catalyst could improve the volume expansion ratio and reduce water retention of PU foams. It was found that Moso bamboo charcoal (*Phyllostachys pubescens*) and China fir wood particle (*Cunninghamia lanceolate*) composites with setting densities of 500 and 600 kg/m^3^ can be prepared from optimized castor oil-based PU foam adhesive at 100 °C for 5 min under a pressure of 1.5 MPa. Increasing the amount of bamboo charcoal decreases the equilibrium moisture content, water absorption and internal bonding strength of the composite. Notably, bamboo charcoal composite exhibits excellent dimensional stability. The optimized density and bamboo charcoal percentages of the composite were 500 kg/m^3^ and 50–100%, respectively. The castor oil-based PU composites containing bamboo charcoal fulfilled the CNS 2215 standards for particleboard. This dimensionally stable, low-density bamboo charcoal composite has high potential to replace current indoor building materials.

## 1. Introduction

Biomass is an abundant renewable resource that differs from dwindling petroleum-based materials because the main components are hydrocarbons, which can be converted to industrial raw materials. A primary goal is to produce biologically-based chemicals directly from biomass, and thus a bio-refinery is a facility that uses biomass raw materials for bio-based products [1]. Biomass has great potential to help shift consumption towards sustainability in chemicals [2], fuels [3] and other materials [4]. Liquid biomass, such as plant oil, is an excellent candidate to efficiently produce bio-based chemicals, and plant oils have been used as a commercial source of biomass-derived polyols for polyurethane (PU) [5]. Currently, PU resin with a urethane linkage molecular structure is one of the most important polymers for industrial products.

Composites combine a fiber and a resin to prepare a matrix with significantly different physical or chemical properties, and PU resin has been used to manufacture lignocellulosic composites. Most previous studies have focused on woody composites, finding that PU and lignocellulosic fibers develop an interfacial interaction for a successful reinforced matrix [6,7,8]. Fiorelli et al. (2012) and El-Shekeil et al. (2012) used a similar processing procedure, where the lignocellulosic fibers were mixed with the PU resin in a mold and then hot-pressed [6,8]. Bamboo charcoal is produced from bamboo, which is a fast-growing speed plant. Bamboo charcoal has many attractive properties, such as a high absorption of harmful gases, humidity regulation, emission of far infrared rays, and high thermal stability, making it an excellent candidate for an indoor building material [9,10,11]. Dimensionally stable and low-density (400–600 kg/m^3^) composites are desired to replace conventional plywood panels. Low-density particleboards are also more dimensionally stable [12]. Bio-based PU foam adhesives from castor oil and PMDI were used to reduce the density. The properties of bio-based PU foams prepared with various dosages of blowing agent and catalyst were measured to determine the optimal conditions for their preparation. 

Castor oil, a non-food plant oil, contains hydroxyl groups, and thus can be used directly as a polyol in chemical industries. Hence, castor oil-based PU resins are an active area of research because of their universal availability, potential biodegradability and low price [13]. PU resin has been used as adhesive, especially in woodworking, and recent studies have focused on castor oil-based PU adhesives. Somani (2003) showed that castor oil-based PU adhesives prepared from aromatic isocyanate have good adhesion strength and chemical resistance [14]. Fiorelli (2012) used a castor oil-based PU adhesive to prepare coconut fiber-based particulate composite [6]. Cravo et al. (2015) used cement packaging residues to manufacture particleboards with castor oil-based PU resin [15]. Tenorio-Alfonso et al. (2017) prepared castor oil-based PU adhesives with cellulose acetate for bonding wood [16]. Hejna et al. (2017; 2008) used crude glycerol and castor oil-based polyol to produce rigid PU foams [17,18]. These results show castor oil can be used for PU adhesive studies. On the other hand, solvents and formaldehyde are already used in a variety of adhesives. However, these low-molecular-weight organic compounds are volatile organic compounds (VOCs), which are major air pollutants, especially for indoor environments [19]. Moreover, a previous study indicated solventless castor oil-based PU adhesive joints showed peeling strength values 75% higher than a solvent-based commercial adhesive [20]. Thus castor oil-based PU resin can be an eco-friendly, bio-based adhesive. However, there are limited studies on the use of bio-based PU resin in bamboo charcoal composites made with a solvent-free process.

This study develops a solvent-free method and hot-pressing process to prepare low-density bamboo charcoal-PU resin composites that also increases forestry resource utilization. Bamboo charcoal, small-diameter timbers and castor oil-based PU resins were used for the composite. Castor oil is a renewable resource and can be used as a polyol in industry. The water absorption, hygroscopicity, dimensional stability and internal bond strength of the composites were investigated.

## 2. Materials and Methods

### 2.1. Materials

China fir (*Cunninghamia lanceolata*) was obtained from the Hui-Sun Experimental Forest Station (National Chung Hsing University, Taichung, Taiwan) and wood particles were ground to pass through a 2.4-mm screen. Bamboo charcoal was prepared from Moso Bamboo (*Phyllostachys pubesens*) in an earth oven at temperatures ranging from 650 to 800 °C and made in Pinglin, Taiwan. The bamboo charcoal was ground to pass between 2.4–3.4 mm diameter screens. The wood and bamboo charcoal were dehydrated in an oven at 105 °C for 12 h. Castor oil was purchased from First Chemical Co., Ltd., Taipei, Taiwan. Potassium hydroxide (KOH) and pyridine and acetic anhydride were reagent-grade chemicals used without pretreatment for measurements of acid and hydroxyl value. KOH was received from Union Chemical Works Ltd., Hsinchu, Taiwan. Pyridine and acetic anhydride were purchased from Shimakyu’s Pure Chemicals, Osaka, Japan. PMDI with a NCO content of 28.9% was used as an isocyanate compound and was purchased from An Fong Develop Co., Ltd., Taichung, Taiwan. Organosiloxane and dibutyltin dilaurate were used as a silicone polyether surfactant and catalyst, respectively. Organosiloxane (DABCO DC5388) and dibutyltin dilaurate were purchased from Ya Chung Industrial Co., Ltd., Taipei, Taiwan and An Fong Develop Co., Ltd., Taichung, Taiwan, respectively. Distilled water was used as the chemical blowing agent from our laboratory.

### 2.2. Properties of Castor Oil and PMDI

Castor oil and PMDI were used directly in the experiments. The acid and hydroxyl value of castor oil and PMDI of NCO% were measured by standard methods [21]. NCO% was calculated as follows: NCO content = Weight of NCO group/Weight of sample × 100. Moisture content of castor oil was measured by Metrohm 703 Ti Stand. Castor oil and PMDI were diluted with THF and deposited on KBr tablets for FT-IR analysis. The FT-IR spectra were measured using a Fourier transform infrared spectrometer (Perkin Elmer Spectrum 100, Waltham, MA, USA), with a diffuse reflectance accessory, and a deuterated triglycine sulfate detector. The spectra were measured within the wavenumber range of 4000–400 cm^−1^ at a resolution of 4 cm^−1^.

### 2.3. Preparation of PU Resins 

To prepare the PU resins, molar ratios of the functional group of NCO for isocyanate to OH for castor oil were set at 1.5. Organosiloxane, dibutyltin dilaurate and water were added as the surfactant, the catalyst, and blowing agent, respectively, with the formulation shown in Table 1. At room temperature, castor oil, with the blowing agent, surfactant and catalyst, was mixed for 30 s at 200 rpm. The isocyanate was then added and stirring continued for 30 s at 200 rpm by the cup test method [22]. Following previous studies [22,23], the cream time, end of rise time and tack-free time of mixtures and volume expansion were recorded during the free rise foaming process.

### 2.4. Characterization of PU Resins

#### 2.4.1. FTIR Analysis

PU resins were ground to pass through a 150-μm screen and water was removed by vacuum oven drying at 40 °C overnight. The samples were mixed with KBr powder at a weight ratio of 1:100. The subsequent methods used were the same as those in Section 2.2.

#### 2.4.2. Density, Water Absorption and Water Retention

The method used for water absorption and water retention of the PU foams followed a previous study [21]. PU foam specimens of 2 × 2 × 2 cm^3^ were used for density and water immersion testing. The density was calculated by the weight and the volume of the foams, with average values reported. The water immersion testing was carried out by dipping the specimens into de-ionized water and measuring the degree of water absorption and water retention after 7 days at room temperature. Water absorption and water retention were calculated as follows: Water absorption (%) = (*W*_1_ − *W*_0_)/*W*_0_ × 100 and Water retention (g/cm^3^) = *W*_1_/*V*_0_; where *W*_0_, *W*_1_ and *V*_0_ denote initial weights, wet weights and volumes of PU foams, respectively.

#### 2.4.3. Weight Retention

The measurement of weight followed that described by a previous investigation [24]. PU foam specimens with dimensions of 1 × 1 × 1 cm^3^ were used to measure weight retention after water immersion. The specimens were immersed in 600 mL of water at 50 °C for 1 h followed by vacuum oven drying at 60 °C and calculation of weight retention. Weight retention was calculated as: Weight retention (%) = (*W*_3_ − *W*_2_)/*W*_2_ × 100; where *W*_2_ and *W*_3_ denote initial and oven-dried weights of PU foams. 

Five specimens were tested for each experiment.

### 2.5. Preparation of Bamboo Charcoal and/or Wood Composites

PU resins were prepared using castor oil with PMDI with a NCO/OH molar ratio of 1.5. The amounts of organosiloxane, dibutyltin dilaurate and water were 4 wt % of castor oil. The PU resin was mixed with bamboo charcoal and/or wood particle or both at a 3:2 weight ratio. Percentages of bamboo charcoal particles were set to 100, 75, 50 or 0%. Densities of the composites were set to 500 or 600 kg/m^3^. Composition of bamboo charcoal and/or wood composites are shown in Table 2. To manufacture the composites, castor oil, surfactant, and catalyst were mixed and stirred, and then the isocyanate and bamboo charcoal and/or wood particles were added and stirred thoroughly again. The reactive mixture was put into a metal mold 15 × 15 × 1.2 cm^3^, and the samples were cured at 100 °C for 5 min at a pressure of 1.5 MPa.

### 2.6. Characterizations of Bamboo Charcoal and/or Wood Composites

Bamboo charcoal and/or wood composites of 5 × 5 × 1.2 cm^3^ were used for all testing. Composites were held at 70% relative humidity (RH) and 27 °C until weight stability. Then equilibrium moisture content was determined using an oven drying method at 103 °C until an approximately constant weight was measured. Density and water immersion testing used the same method as that described for PU foams. Thickness swelling and internal bond strength were measured following the CNS2215:2017 standard [25]. Briefly, the specimens were immersed in water at 20 °C for 24 h, followed by drying and calculating the thickness. For internal bonding, specimens were glued to a steel block with a hot melt adhesive and evaluated with a universal strength testing machine (Shimadzu UEH-10, Kyoto, Japan) using a loading speed of 2 mm/min^−1^ in the direction perpendicular to the panel surface.

### 2.7. Statistical Analyses

Results for PU resin and composites are shown as mean and standard deviation. Statistical analysis was performed using SPSS software version 20 (SPSS Inc., Chicago, IL, USA). Tukey’s multiple range test was used to find the statistical significance (*P* = 0.05) between pairs.

## 3. Results and Discussion

### 3.1. Foaming Behavior of PU Resins

Acid value and hydroxyl value are related to the degradation degree of the –COOH and –OH groups from castor oil. To prepare PU resins, castor oil is an excellent candidate because it is rich in –OH groups. The acid value and hydroxyl value of castor oil were 1.8 and 193.8 –KOH/g, respectively, which was similar to a previous study [26]. The moisture content of castor oil was 0.12%. In addition, isocyanate contains –NCO groups that could react with –COOH and –OH groups to yield a urethane linkage, and the –NCO of PMDI is 28.9%. A previous investigation indicated that the molecular weight of castor oil is 928 g/mol [27]. Results for the raw materials indicated the functional groups of raw materials agreed with the prepared PU resins.

Our previous study demonstrated that increasing the NCO/OH ratio would decrease tack-free time and weight during the leaching test. For the preparation of the composite, 1 min was required before the reactive mixture could be put into a metal mold. Kong (2011) indicated that a NCO/OH ratio of 1.5 resulted in the best compromise of adhesive joint strength and cohesion of bio-based PU wood adhesives [28]. Therefore, PU resins with a NCO/OH ratio of 1.5 have an optimal operation time and network structure [29]. Table 3 shows the foaming behavior of PU resins with respect to the reactivity of castor oils and PMDI by an exothermic process. Increasing the blowing agent can shorten cream time, end of rise time and tack-free time. The results indicate that increasing the blowing agent can help the rate of expansion and gelation rate because water is the chemical blowing agent [30]. On the other hand, catalyst concentration did not yield a significant foaming behavior. The dosage-effect relationship of the blowing agent and catalyst was similar to that of a previous study [31]. The volume expansion of PU resins is greater than 250%. These phenomena may be due to –OH groups of castor oils reacting with –NCO groups of PMDI.

### 3.2. Density, Water Absorption and Resistance of PU Resins

**Figure 1:**
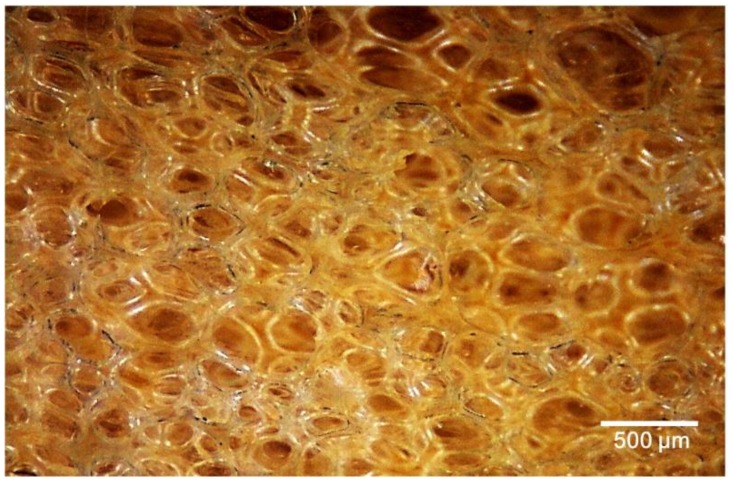
Optical micrograph of PU foam P-4-4.

This investigation used castor oil as a raw material for polyol, PMDI as isocyanate, water as a blowing agent and organosiloxane as a surfactant. In our previous study, the amount of catalyst was optimized and fixed as 4 parts by weight [29]. Table 4 displays the water absorption and resistance of PU resins prepared with castor oil. The amount of catalyst and blowing agent was adjusted by 2–4 parts by weight of castor oil. With increasing blowing agent, the density of PU resins increased, while their water absorption decreased significantly. On the other hand, water retention of PU resins slightly decreased with increasing surfactant. All weight retentions of PU resins are similar and higher than 98.5%. Therefore, the network structure of PU resins is formed because of a complete crosslink reaction in a well-blended mixture of raw materials. These results demonstrate that P-4-4 has low water retention and high volume expansion. The image obtained by the optical microscopy of the cross-section of P-4-4 is shown in Figure 1. This indicates that P-4-4 has a uniform periodic cellular structure and is an open-cell porous PU foam. Based on the results of the evaluation, P-4-4 can be considered the optimized PU foam for preparing the bamboo charcoal/wood composite.

### 3.3. FTIR Spectra of PMDI, Castor Oil and PU Resins

**Figure 2:**
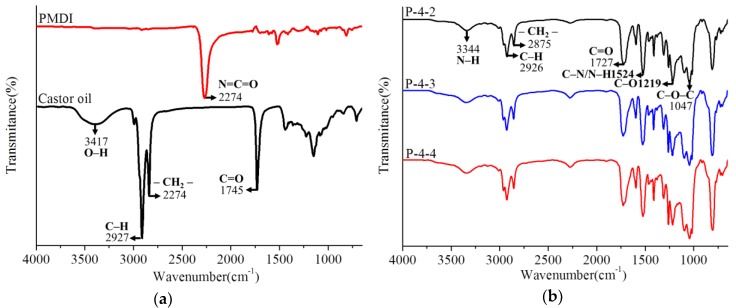
FTIR spectra of poly(4,4’-methylene diphenyl isocyanate) (PMDI), castor oil (**a**) and PU resins (**b**).

The FTIR spectra of castor oil and PMDI are shown in Figure 2a, where 2274 cm^-1^ is assigned to the –NCO group of isocyanate PMDI. Castor oil shows a broad hydroxyl band between 3640 and 3210 cm^−1^, and a typical triglyceride ester methyl/methylene and carbonyl at 2927/2274 and 1745 cm^−1^ [32]. Figure 2b shows the FTIR spectra of PU resins P-4-2, P-4-3 and P-4-4. The spectrum for the PU resins derived from castor oil and PMDI shows a very similar pattern, indicating that the amount of catalyst has no effect on the structure. The broad band around 3344 cm^−1^ was attributable to the amine group (NH stretch) and existence of the hydroxyl group. Absorption bands were assigned to methyl (2926 cm^−1^) and methylene (2853 cm^−1^) groups from castor oil. The very weak absorbance band between 2200–2300 cm^−1^ was due to the –NCO group. Because PMDI can be a wood adhesive, the –NCO group of PMDI and its reaction with both the –OH group and water in the PMDI/wood mixtures was also demonstrated. Therefore, residual isocyanates were attributed to networks between resin and fillers [33,34]. The absorption band at 1727 cm^−1^ was due to carbonyl stretching vibration (amide I band) of the urethane linkages [35]. The band at 1524 cm^−1^ was assigned to N–H and C–N bending vibration of urethane linkages [36]. The two bands 1219 and 1047 cm^−1^ were attributed to C–O stretching vibration and C–O–C stretching vibration [37]. The absorption bands at 1727, 1524 and 1219 cm^−1^ demonstrated urethane linkages that were formed between castor oil and PMDI. Those results are similar to a previous study that demonstrated polymerized urethanes based on castor oil and 4,4’-methylene diphenyl isocyanate (MDI), as analyzed by FTIR [38].

### 3.4. Appearance and Densities of Bamboo Charcoal/Wood Composite

**Figure 3:**
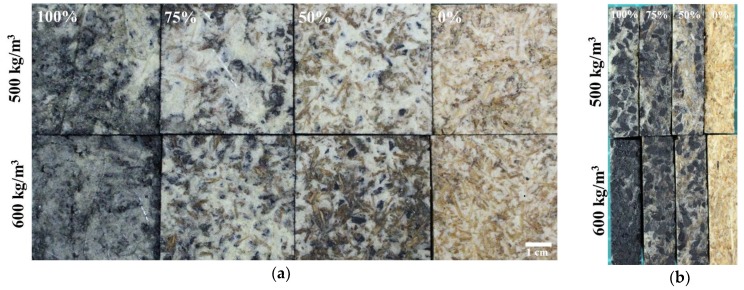
Front view (**a**) and side view (**b**) of charcoal and/or wood composites.

**Figure 4:**
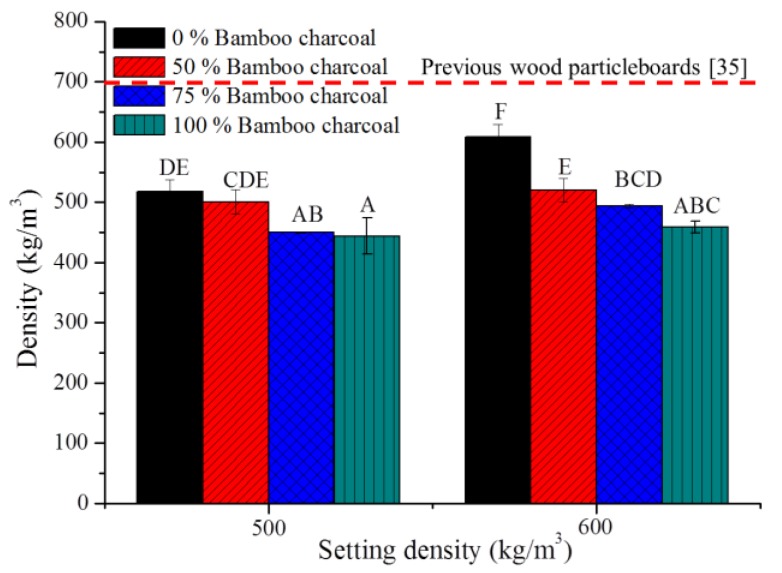
Densities of bamboo charcoal and/or wood composites. The red dotted line indicates the density of pine wood particleboards prepared with castor oil-based PU adhesive from a previous study [39]. Means with different capital letters indicate significant differences between bamboo charcoal composites using Tukey’s multiple range tests at *p* < 0.05.

The previous results show that P-4-4 with high water resistance and low-density is the optimal condition for a charcoal and/or wood composite. Originally, the PU resin was mixed with particles at a 1:1 weight ratio, but the composite collapsed, indicating that the PU resin could not form a stable foam structure with good adhesive property. After modifying the ratio of PU resin and particles to 3:2, bamboo charcoal and/or wood composites were prepared successfully, as shown in Figure 3. Bamboo charcoal, wood particles and PU resin were homogenously distributed in the low-density composites. Figure 4 shows the densities of bamboo charcoal and/or wood composites are between 444 and 609 kg/m^3^. This result shows that densities of wood composites match the setting densities. On the other hand, densities of bamboo charcoal and/or wood composites decreased significantly when their percentage of charcoal was increased. Densities of P-4-4 bamboo charcoal and wood are 22.2, 690 and 360 kg/m^3^. Therefore, the bamboo charcoal composite occupies more space than the wood composite and can form PU foam in the preparation process. Wechsler et al. (2013) indicated the densities of the macadamia shell and pine wood particleboards prepared with castor oil-based PU adhesive were 987 and 691 kg/m^3^ [39]. Foaming phenomenon was observed in the preparation of the composites. The gas formed from foams can help join bamboo charcoal and wood together and reduce the density of the composite. These results indicate that castor oil-based PU resin could produce low-density composites in this formulation.

### 3.5. Hygroscopicity and Water Absorption of Bamboo Charcoal and/or Wood Composites

**Figure 5:**
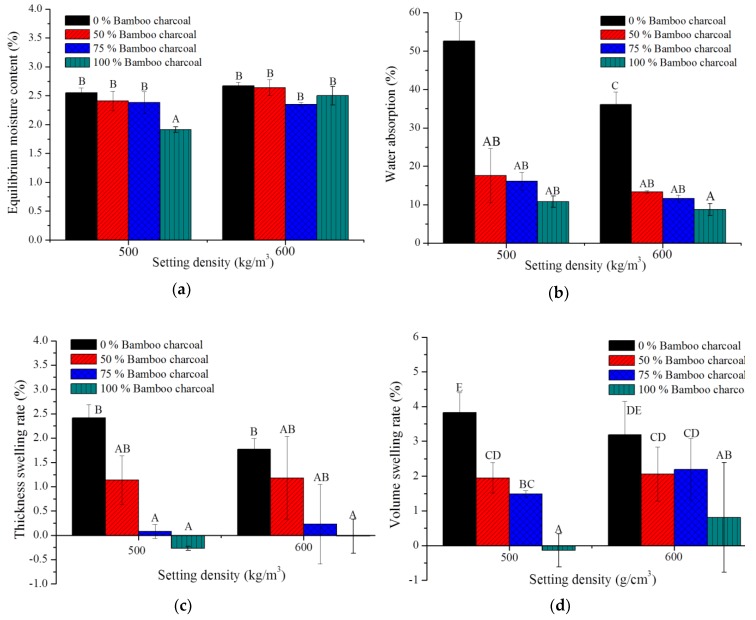
Equilibrium moisture content (**a**), water absorption (**b**), thickness (**c**) and volume swelling rate (**d**) of bamboo charcoal and wood composites. Means with different capital letters indicate significant differences between bamboo charcoal composites using Tukey’s multiple range tests at *p* < 0.05.

Lignocellulose materials hold bound water and free water in their cell walls and cavities because of the surface hydroxyl groups. Hygroscopicity influences dimensional stability, mechanical properties, thermal properties and durability [40]. Figure 5a shows the equilibrium moisture content of bamboo charcoal and/or wood composites to be lower than 2.67%. With the density at 500 kg/m^3^, the charcoal and/or wood composite is significantly lower than other specimens. The previous investigation indicated the equilibrium moisture content of raw straw particleboard with methylene diphenyl diisocyanate and urea formaldehyde adhesive to be 7.65% and 7.97% at 65% RH and 23 °C. These results are due to low hydroxyl groups binding water on the surface of the composites [41]. Figure 5b shows the water absorption of bamboo charcoal and/or wood composites, which are substantially lower than wood composites. The water absorptions of bamboo charcoal composites are 10.8% and 8.8% with setting densities of 500 and 600 kg/m^3^, and the water absorption of PU resins decreased slightly with increased bamboo charcoal content. Cravo et al. [15] found water absorption of castor-based resin paper residue particleboards to be 70.4% and 56.8% with densities of 500 and 600 kg/m^3^. The water absorption of conventional particleboard with urea-formaldehyde adhesive is 31–41% with densities of ca. 700 kg/m^3^ [42]. Bamboo charcoal and castor oil-based PU resin are highly hydrophobic [5,43]. These results demonstrate that bamboo charcoal-based composites manufactured from castor oil-based PU resin can help reduce water absorption and hygroscopicity of materials.

### 3.6. Dimensional Stability of Bamboo Charcoal and/or Wood Composites

For traditional lignocellulose composition, dimensional stability is a basic requirement. Figure 5c,d show the thickness and volume swelling of bamboo charcoal and/or wood composites after 24 h of water soaking. The results indicate that thickness and volume swelling decreased significantly with bamboo charcoal added to the composite. Thickness swelling of bamboo charcoal and wood composites is less than 2.41%, and volume swelling ranged from 3.83% to –0.14%. Notably, the thickness swelling values of bamboo charcoal composites are –0.27% and –0.02% with densities of 500 and 600 kg/m^3^. Fabiyi et al. (2011) and Fuentes Talavera et al. (2007) showed the thickness swellings of wood and bagasse plastic composite with high-density polyethylene ranged from 1.68% to 5.82% and 6.9% to 15.3% after 1 day of water soaking [44,45]. Que et al. (2007) indicated thickness swelling of urea-formaldehyde conventional particleboard is 13.8% and 16.3% [42]. This result suggests that bamboo charcoal composites are much more dimensionally stable than other wood composites, highlighting the importance of bamboo charcoal and castor oil-based PU resin in the formulation. The bamboo charcoal-based composite has high dimensional stability that is attributable to its hydrophobicity and low-density.

### 3.7. Internal bond strength of bamboo charcoal and wood composites

**Figure 6:**
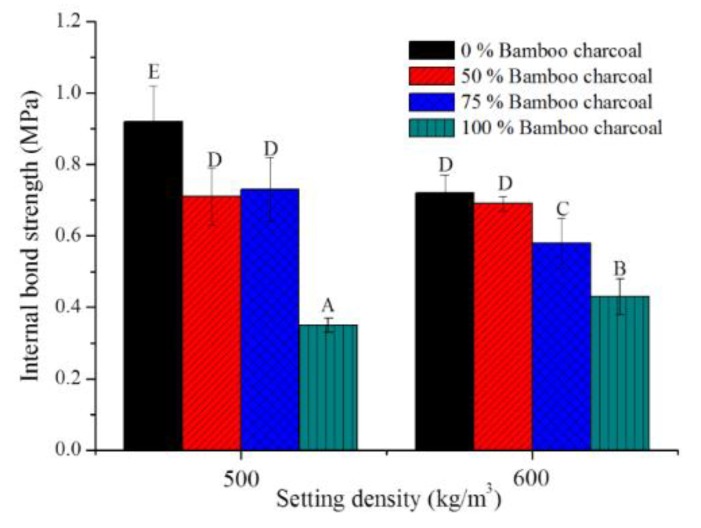
Internal bond strengths of bamboo charcoal and wood composites. Means with different capital letters indicate significant differences between bamboo charcoal composites using Tukey’s multiple range tests at *p* < 0.05.

The Chinese National Standards, CNS2215:2017 [25], Type 18, 13 and 8 minimum requirements for internal bond strength are 0.3, 0.2 and 0.15 MPa, respectively. Figure 6 shows the internal bond strength of bamboo charcoal and wood composites is from 0.35 to 0.92 MPa. Increased wood content could increase the internal bonding strength. Que et al. (2007) and Wang et al. (2007) showed the internal bond strength of conventional particleboard with urea-formaldehyde and PMDI to be 0.88 and 0.56 MPa [42,46]. Internal bond strength of all composites can fit the requirements for Type 18 of CNS2215:2017, similar to other composites. Type 18 of CNS2215:2017 represents a particleboard standard with the required internal bond strength of >0.3 MPa. For conventional particleboards, internal bonding strength and density are highly correlated in direct proportion [47], and bamboo charcoal composites are similar. The internal bond strength of bamboo charcoal and wood composites with a density of 500 kg/m^3^ was slightly higher than those with a density of 600 kg/m^3^. These results suggest castor oil-based PU resin performs well in adhering wood and bamboo charcoal.

## 4. Conclusions

PU resins were prepared by blending castor oil with PMDI at a molar ratio of 1.5 for NCO/(COOH+OH). Water, organosiloxane and dibutyltin dilaurate were added as blowing agent, surfactant and catalyst, respectively. The blowing agent and catalyst should be adjusted to optimize the properties as castor oil was used in the manufacturing of PU resins. The results demonstrate that 4% catalyst and blowing agent should be added to minimize water absorption and maximize volume expansion as castor oil was used in the manufacturing of PU foams. The composites had a mixture of PU resin and bamboo charcoal and/or wood particle with a weight ratio of 1.5/1.0 by hot-pressing. The properties of composites made under different conditions were investigated. Comparing different ratios of wood particle and bamboo charcoal, using solely bamboo charcoal as the raw material had the best dimensional stability. The bamboo charcoal and/or wood composite with 500 kg/m^3^ had higher internal bonding strength than particleboards with 600 kg/m^3^. The internal bonding strength of bamboo charcoal and wood particleboards decreased slightly with a higher bamboo to charcoal ratio for the particleboards. The internal bonding strength of 50% bamboo charcoal composite was equal to particleboard entirely composed of wood. Future functional study of this product can assist its application in the forest product and building material industries.

## Figures and Tables

**Table 1:** Polyurethane (PU) resins formulation (parts by weight).

Code^1^	PMDI	Castor oil	Surfactant	Blowing agent	Catalyst
P-2-2	67.1	100	4	2	2
P-2-3	67.1	100	4	2	3
P-2-4	67.1	100	4	2	4
P-3-2	67.1	100	4	3	2
P-3-3	67.1	100	4	3	3
P-3-4	67.1	100	4	3	4
P-4-2	67.1	100	4	4	2
P-4-3	67.1	100	4	4	3
P-4-4	67.1	100	4	4	4

**^1^P-x-y.** Amounts of blowing agent and catalyst are x and y parts by weight of castor oil.

**Table 2:** Composition of bamboo charcoal and/or wood composites for a dimension of 15 × 15 × 1.2 cm^3^.

Setting densities (kg/m^3^)	P-4-4 (g)	Bamboo charcoal (g)	Wood particle (g)
500	81	0	54
500	81	27	27
500	81	40.5	13.5
500	81	54	0
600	97.2	0	64.8
600	97.2	32.4	32.4
600	97.2	48.6	16.2
600	97.2	64.8	0

**Table 3:** Foaming behavior of the PU resins.

Code	Cream time (s)	End of rise time (s)	Tack-free time (s)	Volume expansion (%)
P-2-2	32	62	90	250
P-2-3	33	62	81	360
P-2-4	31	62	83	500
P-3-2	26	46	58	300
P-3-3	26	48	60	340
P-3-4	29	56	80	380
P-4-2	22	47	54	300
P-4-3	28	47	60	330
P-4-4	23	41	59	350

**Table 4:** Density, water absorption and resistance of PU resins.

Code	Density (kg/m^3^)	Water absorption (%)	Water retention (g/cm^3^)	Weight retention (%)
P-2-2	25.7 ± 0.6^EF^^,1^	43.0 ± 4.5^AB^	0.11 ± 0.01^B^	99.0 ± 0.1^A^
P-2-3	17.8 ± 0.3^B^	61.4 ± 4.1^CD^	0.11 ± 0.01^B^	99.2 ± 0.7^A^
P-2-4	13.3 ± 0.2^A^	88.6 ± 3.7^E^	0.12 ± 0.01^B^	99.0 ± 0.1^A^
P-3-2	25.8 ± 0.4^F^	36.1 ± 2.8^A^	0.09 ± 0.01^AB^	99.2 ± 0.1^A^
P-3-3	24.1 ± 0.8^E^	42.2 ± 3.3^AB^	0.10 ± 0.01^AB^	99.2 ± 0.1^A^
P-3-4	17.8 ± 0.3^B^	65.7 ± 5.5^D^	0.12 ± 0.01^B^	98.3 ± 1.8^A^
P-4-2	25.0 ± 1.4^EF^	33.8 ± 3.5^A^	0.08 ± 0.01^A^	98.6 ± 0.1^A^
P-4-3	21.0 ± 0.7^C^	49.9 ± 6.4^BC^	0.11 ± 0.01^B^	98.5 ± 0.2^A^
P-4-4	22.2 ± 0.3^D^	50.7 ± 6.2^BC^	0.10 ± 0.01^AB^	98.5 ± 0.1^A^

**^1^** Means and standard deviation with different capital letters indicate significant differences between PU resins using Tukey’s multiple range tests at *p* < 0.05.
